# A Deep Learning Approach for Dengue Fever Prediction in Malaysia Using LSTM with Spatial Attention

**DOI:** 10.3390/ijerph20054130

**Published:** 2023-02-25

**Authors:** Mokhalad A. Majeed, Helmi Zulhaidi Mohd Shafri, Zed Zulkafli, Aimrun Wayayok

**Affiliations:** 1 Department of Civil Engineering, Faculty of Engineering, Universiti Putra Malaysia (UPM), Serdang 43400, Selangor, Malaysia; 2 Geospatial Information Science Research Centre (GISRC), Faculty of Engineering, Universiti Putra Malaysia (UPM), Serdang 43400, Selangor, Malaysia; 3 Department of Biological and Agricultural Engineering, Faculty of Engineering, Universiti Putra Malaysia (UPM), Serdang 43400, Selangor, Malaysia

**Keywords:** dengue fever, LSTM, spatial attention, temporal attention, Malaysia

## Abstract

This research aims to predict dengue fever cases in Malaysia using machine learning techniques. A dataset consisting of weekly dengue cases at the state level in Malaysia from 2010 to 2016 was obtained from the Malaysia Open Data website and includes variables such as climate, geography, and demographics. Six different long short-term memory (LSTM) models were developed and compared for dengue prediction in Malaysia: LSTM, stacked LSTM (S-LSTM), LSTM with temporal attention (TA-LSTM), S-LSTM with temporal attention (STA-LSTM), LSTM with spatial attention (SA-LSTM), and S-LSTM with spatial attention (SSA-LSTM). The models were trained and evaluated on a dataset of monthly dengue cases in Malaysia from 2010 to 2016, with the task of predicting the number of dengue cases based on various climate, topographic, demographic, and land-use variables. The SSA-LSTM model, which used both stacked LSTM layers and spatial attention, performed the best, with an average root mean squared error (RMSE) of 3.17 across all lookback periods. When compared to three benchmark models (SVM, DT, ANN), the SSA-LSTM model had a significantly lower average RMSE. The SSA-LSTM model also performed well in different states in Malaysia, with RMSE values ranging from 2.91 to 4.55. When comparing temporal and spatial attention models, the spatial models generally performed better at predicting dengue cases. The SSA-LSTM model was also found to perform well at different prediction horizons, with the lowest RMSE at 4- and 5-month lookback periods. Overall, the results suggest that the SSA-LSTM model is effective at predicting dengue cases in Malaysia.

## 1. Introduction

Dengue fever is a viral illness transmitted by mosquitoes that are found in tropical and subtropical regions around the world [1]. It is characterized by symptoms such as fever, headache, muscle and joint pain, and rash. In severe cases, dengue fever can lead to complications such as dengue hemorrhagic fever, which can be life-threatening. Dengue fever is a major public health concern; as of December 31st, 2022, a total of 4,110,465 cases of dengue and 4099 fatalities have been reported [2]. It imposes significant economic and social costs, including loss of productivity, healthcare expenses, and social disruption. Several factors contribute to the spread of dengue fever, including the presence of mosquito breeding sites, population density, and environmental factors such as temperature and rainfall. Understanding these risk factors can help in the development of strategies to prevent and control dengue fever outbreaks.

Dengue fever is a significant public health concern in Malaysia, with outbreaks occurring regularly in the country [3]. According to the World Health Organization (WHO), Malaysia has one of the highest rates of dengue fever in the Western Pacific region, with an estimated 330,000 cases reported in 2020. In Malaysia, the government has implemented several measures to control and prevent dengue fever outbreaks, including vector control (e.g., removing mosquito breeding sites), public education campaigns, and vaccination. Despite these efforts, dengue fever continues to be a major public health concern in Malaysia.

Machine learning approaches can be used to model and predict the risk of dengue fever outbreaks, which can inform the development of prevention and control strategies. Long short-term memory (LSTM) is a type of recurrent neural network that is well suited for modeling time series data, such as data on the incidence of dengue fever over time [4]. LSTM networks can learn long-term dependencies in the data by using gates that control the flow of information within the network. This allows LSTM networks to effectively capture patterns that may span multiple time steps, which can be useful for predicting future outcomes. Spatial attention and temporal attention are two types of attention mechanisms that can be used in LSTM networks for dengue fever prediction or other tasks [5]. Spatial attention is a mechanism that allows a model to weigh different features differently when making predictions. In the context of dengue fever prediction, spatial attention could be used to prioritize certain features that are more relevant to the prediction task, such as environmental factors or human population density. By weighing these features more heavily, the model can better focus on the most important information when making predictions. Combining LSTM and spatial attention can improve the accuracy of dengue fever prediction by allowing the model to effectively capture long-term patterns in the data and focus on the most relevant features when making predictions. This can lead to more accurate and robust predictions, which can be useful for public health efforts to prevent and control dengue fever outbreaks. On the other hand, temporal attention is more suitable for dengue forecasting tasks and has been discussed in several studies [5]. 

This paper develops and validates an LSTM model with spatial attention for dengue prediction. The remaining part of this paper is organized as follows: Section 2 discusses the previous studies and highlights the need for the new proposed model, Section 3 describes the data used and the proposed methodology, and Section 4 presents the results of several experiments conducted in the research. This section also compares the results of the proposed model with several benchmark models. Finally, Section 5 presents the conclusion of the research and provides the limitations of the proposed models and directions for future work. 

## 2. Literature Review 

Dengue fever is a significant public health concern in the world and is projected to worsen in the future due to climate change. The development of an early-warning system for dengue fever has been identified as a priority health adaptation measure in many countries. Several studies have explored the use of machine learning and deep learning for dengue prediction. Several machine learning and statistical models have been developed for dengue prediction. These models typically use data on various environmental and meteorological factors, such as temperature, humidity, and rainfall, to predict the dengue fever cases in a given area. Other factors that may be included in the model include population density, land use, and the presence of breeding sites for *Aedes* mosquitoes. Sarma et al. [6] provide an overview of the various machine learning techniques that have been used for dengue prediction, including decision trees, artificial neural networks, support vector machines, and random forests. The authors also discuss the strengths and limitations of these techniques. The most common models are support vector machines [7], decision trees [8], random forests [9], and neural networks [10]. In addition, several studies have compared those machine learning models for dengue prediction in different contexts [11,12,13,14].

In recent years, deep learning models such as LSTM and convolutional neural networks (CNNs) have been developed for dengue prediction. Doni and Sasipraba [15] used an LSTM model to predict the impact of dengue cases in India. They found that the model had an 89% accuracy in forecasting dengue infection and 81% accuracy in forecasting deaths. They observed that this accuracy level resulted from the increase in the number of iterations contributing to the decrease in the root mean squared error (RMSE). Xu et al. [16] developed a timely and accurate forecasting model for dengue fever cases in China using LSTM. The researchers compared the performance of LSTM models with other previously published models when predicting dengue fever cases one month into the future. The results of the study showed that the LSTM model had superior performance in predicting dengue fever cases, reducing the average RMSE of the predictions by 12.99% to 24.91%. The LSTM model also had improved performance in predicting dengue fever cases during outbreak periods, reducing the average RMSE of the predictions by 15.09% to 26.82% compared to other candidate models. In a separate study, Saleh and Baiwei [17] explored the use of LSTM time series forecasting for predicting dengue cases in Malaysia. They compared the performance of LSTM with support vector regression (SVR) and found that LSTM performed better in forecasting dengue cases. The authors also found that the inclusion of environmental factors, such as temperature and humidity, improved the accuracy of the LSTM model. Saleh and Baiwei [17] investigated the use of LSTM to predict dengue cases in Malaysia. The researchers developed an LSTM model and compared it to an SVR model using a dengue dataset consisting of weather and climate data. The results of the study showed that the LSTM model performed better than the SVR model, with an R2 score of 0.75 and a mean absolute error (MAE) score of 8.76. The LSTM model was able to capture long-term dependencies and trends in the rise and fall of dengue cases, which makes it a promising tool for predicting dengue cases without relying on forecasted weather data.

Other studies have also investigated the use of deep learning for dengue prediction. For example, Alfred and Obit [18] developed a hybrid model combining a CNN and LSTM for dengue prediction in Iran. The model was trained using meteorological and dengue incidence data from 2010 to 2016 and was able to achieve high accuracy in predicting dengue cases in the test dataset from 2017 to 2018. In contrast, Mussumeci and Coelho [19] compared the performance of an LSTM model with a random forest regression model for forecasting dengue incidence in Brazil. They found that the LSTM model had the highest performance in predicting the future incidence of dengue in cities of different sizes. Similarly, Nadda et al. [20] found that the LSTM model with a fully connected neural network had better accuracy than the cross-entropy loss function for detecting dengue fever using symptom text. Khaira et al. [21] also compared the performance of an LSTM model with a SARIMA model for forecasting dengue incidence in Jambi, Indonesia, and found that both models performed relatively well. 

Several studies have found that LSTM models can be effective in predicting dengue fever incidence. LSTM models are a type of recurrent neural network that is particularly well suited for handling time series data and capturing long-term dependencies in the data. These characteristics make LSTM models a promising tool for forecasting dengue fever incidence. Some studies have also found that using transfer learning or incorporating other input data, such as climatic conditions and population data, can improve the accuracy of the LSTM models. It is also worth noting that different studies have used different evaluation metrics, such as MAE, RMSE, and G-mean, to assess the performance of the LSTM models, so it is difficult to directly compare the results of these studies. It is also important to note that LSTM models are dependent on the quality and representativeness of the data used to train them, and their performance may vary depending on the specific context and data available.

Moreover, Nadda et al. [22] found that LSTM is effective in capturing long-term dependencies in time series data and has shown superior performance in predicting dengue cases compared to other models. LSTM models have been combined with other machine learning techniques, such as fully connected neural networks or feature selection methods, to further improve their performance. It is also noted that transfer learning, a method of fine-tuning a pretrained model on a new dataset, can be used to improve the generalization ability of LSTM models in areas with fewer dengue cases. However, some studies suggest that attention mechanisms can improve the performance of LSTM models for dengue prediction. Attention mechanisms allow LSTM models to weigh different input variables differently, depending on their importance for prediction. This can be particularly useful in dengue prediction, as different meteorological and epidemiological factors may have different impacts on dengue transmission. Nguyen et al. [5] developed a prediction model for dengue fever in Vietnam using deep learning models, specifically CNN, transformer, LSTM, and attention-enhanced LSTM (LSTM-ATT). These models were trained using lagged dengue fever incidence data and meteorological variables (such as temperature, humidity, rainfall, evaporation, and sunshine hours) from 20 provinces in Vietnam from 1997 to 2013. The models were then evaluated using data from 2014 to 2016 by measuring their RMSE and MAE. The results of the study showed that the LSTM-ATT model performed the best, with average ranks of 1.60 for the RMSE-based ranking and 1.95 for the MAE-based ranking. It was also able to accurately predict dengue fever incidence and outbreak months up to 3 months ahead, although its performance slightly decreased compared to short-term forecasts. These findings highlight the importance of attention mechanisms in LSTM models for dengue prediction. Attention mechanisms can help LSTM models identify the most important variables for prediction and to weigh them accordingly, leading to improved performance. 

A limitation of these studies is that they have limited evaluation of the performance of the models for long-term forecasts. Most of the studies used data from a few years to train and evaluate the models, and it is unclear how well the models would perform for longer-term forecasts. Additionally, most of the studies used data from a specific period and region, which may limit the generalizability of the findings to other regions and periods. Further research is needed to determine the generalizability of the findings and to determine the performance of the models for long-term forecasts. These studies suggest that LSTM can be an effective deep-learning model for dengue prediction, particularly when an attention mechanism is incorporated. Further research is needed to determine the optimal implementation of LSTM and attention mechanisms in dengue prediction and to determine the generalizability of these findings to other regions.

## 3. Data and Methodology

### 3.1. Description of Study Area

**Figure 1:**
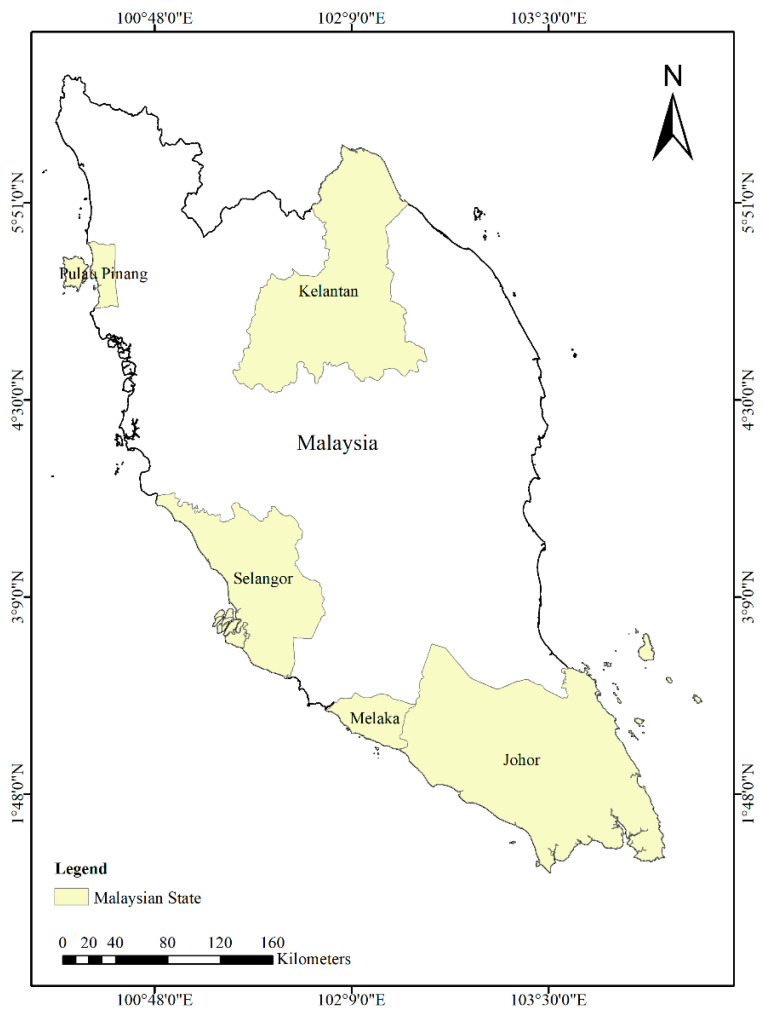
Map of Peninsular Malaysia showing the five states studied in this work.

Malaysia is a Southeast Asian nation that extends across latitudes 1° to 7° N and longitudes 99° to 105° E [23] (Figure 1). It has a total land area of 131,587 square kilometers and is divided into two regions by the South China Sea: Peninsular Malaysia and East Malaysia, which includes the states of Sabah, Sarawak, and Labuan (also known as Malaysian Borneo). Figure 1 illustrates the map of the study area.

The first recorded cases of dengue fever in Malaysia were reported in 1902, but it was not until the 1970s that dengue became a significant public health concern in the country. In 1973, the first major outbreak of dengue occurred [24,25]. Over the past few decades, the number of dengue cases in Malaysia has been steadily increasing. According to recent statistics, the number of cases per 100,000 people in the country has increased from 32 in 2000 to 361 in 2014. The majority of dengue patients in Malaysia are between the ages of 15 and 49, and approximately 80% of cases occur in urban areas (Ministry of Health in Malaysia).

Malaysia’s geographical location and climate make it prone to the spread of infectious diseases such as dengue fever. The country has a humid tropical climate with temperatures ranging from 21 to 32 °C and is home to floodplains, hills, and coastline zones. There are two rainy seasons in Malaysia: The Northeast Monsoon, which lasts from October to March [26], and the Southwest Monsoon, which runs from May to September [27]. April is a transitional month with significant amounts of rainfall [23]. The states of Selangor, Kelantan, Johor, Pulau Pinang, and Melaka are among the areas in Malaysia with the highest incidences of dengue fever. In particular, the state of Selangor alone accounts for 90% of national dengue cases in Malaysia (Ministry of Health in Malaysia). By focusing on the states with the highest incidence rates, the model would be better equipped to identify and account for the unique factors that drive the spread of dengue fever in these regions, such as climate, human population density, and socioeconomic status.

### 3.2. Description of the Dataset

For this research, a dataset consisting of weekly dengue cases at the state level in Malaysia from 2010 to 2016 was obtained from the Malaysia Open Data website. The data were provided by the Ministry of Health and pertain to five states: Selangor, Kelantan, Johor, Pulau Pinang, and Melaka. They include the total number of dengue deaths in each state for each week and have been aggregated into monthly cases for analysis. This dataset offers insight into the prevalence of dengue fever in Malaysia over seven years and can be used to identify trends and patterns in the disease.

**Figure 2:**
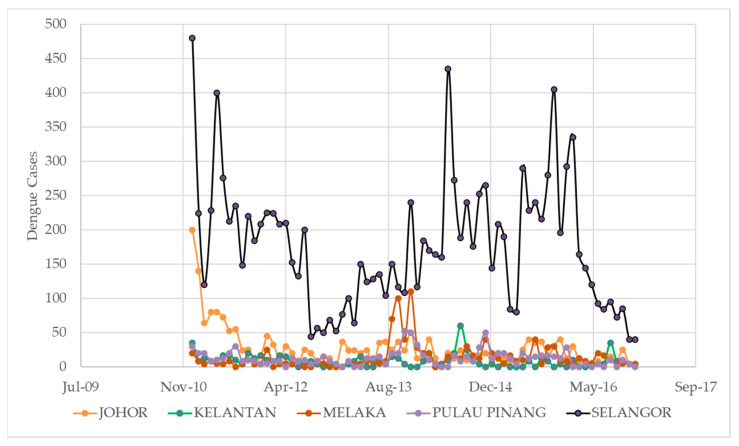
Monthly dengue fever incident cases for five different states in Malaysia from 2011 to 2016.

Figure 2 shows the number of dengue cases per month from 2010 to 2016 for five states in Malaysia: Johor, Kelantan, Melaka, Pulau Pinang, and Selangor. The figure is organized by month and year, with the number of cases for each state listed in the corresponding column. The data show that the number of dengue cases varied greatly from month to month and from state to state, with some states (such as Selangor) consistently having higher numbers of cases compared to others. The minimum number of dengue cases in a month was 0, which occurred in several states at various times throughout the period. The maximum number of cases in a single month was 480, which occurred in Selangor in January 2011. The mean number of dengue cases per month for Johor was 22.8, with a standard deviation of 37.3. For Kelantan, the mean was 8.5 and the standard deviation was 10.6. The mean for Melaka was 15.5 with a standard deviation of 27.8, and for Pulau Pinang, the mean was 14.2 with a standard deviation of 16.4. Finally, for Selangor, the mean was 177.8 with a standard deviation of 116.3.

It is also worth noting that the range of dengue cases per month varied significantly between the different states. For example, the range for Johor was 180 cases (200 in January 2011 to 20 in March 2011), while the range for Selangor was 455 cases (480 in January 2011 to 25 in October 2011). This indicates that the spread of dengue cases was more variable in some states compared to others. Overall, the data suggest that dengue is a complex and multifaceted problem in Malaysia, with the spread of cases being influenced by a variety of factors at the state and local levels. To effectively address the problem, it will likely be necessary to adopt a multipronged approach that takes into account these various factors and seeks to address the root causes of the disease.

**Figure 3:**
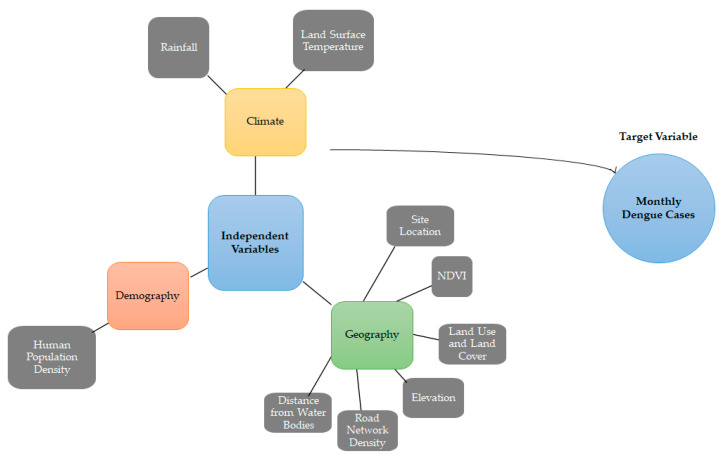
An illustration of the variables used in the dengue prediction models.

In addition, the present study utilizes a dataset encompassing various variables relevant to dengue fever cases, climate, geography, and demographics in the five states of Malaysia from 2011 to 2016 (Figure 3). The dependent variable of the dataset is the number of dengue cases, recorded at the state level with a temporal resolution of monthly. The source of these data is the Malaysia Open Data platform. The climate variables included in the dataset are rainfall and land surface temperature with temporal resolutions of daily and monthly, respectively; both have a spatial resolution of 0.25 degrees. The sources for these data are the CMORPH precipitation dataset and the MODIS dataset. The geography variables in the dataset consist of vegetation index, digital elevation model, land cover, road networks, and water bodies, with spatial resolutions ranging from 0.05 degrees for vegetation index and land surface temperature to 30 arc-seconds for digital elevation model, land cover, road networks, and water bodies. The temporal resolution for the vegetation index is 16 days, and the sources for these data are the MODIS dataset and the Diva GIS platform (using data from the Digital Chart of the World and CGIAR SRTM). The demographic variable included in the dataset is population, recorded at the state level with a yearly temporal resolution and sourced from the Malaysia Open Data platform.

### 3.3. Data Preprocessing

Before analyzing the dengue dataset described earlier, various preprocessing steps were carried out to ensure that the data were in a suitable format and had been properly cleaned and filtered. This process, referred to as data preparation, involved transforming the data from one format to another, as well as registering the datasets to match them geographically and temporally. This was necessary to ensure that all of the data were compatible and could be accurately compared and analyzed.

An important aspect of preprocessing the dengue dataset was the creation of lag variables. These are variables that represent the value of a particular variable at a specific point in the past. In this case, lag variables were created with a time lag of 1 to 6 months ahead. This allows for the analysis of the relationships between variables over time, helping to identify trends and patterns in the data that may not be immediately apparent. Overall, these preprocessing steps were essential in preparing the dengue dataset for analysis and ensuring that the results of the analysis were as accurate and reliable as possible.

### 3.4. Dengue Prediction 

#### 3.4.1. LSTM

Traditional feed-forward neural networks are effective at learning features from data, but they are not well suited for modeling sequential data. Recurrent neural networks (RNNs) are designed to address this limitation by incorporating feedback connections that allow the model to explicitly capture the temporal dynamics of sequential data. RNNs are more biologically plausible than feed-forward neural networks, as they incorporate a memory of previous activations that allows them to learn about the underlying temporal structure of the data. However, RNNs can be prone to issues such as vanishing or exploding gradients, which can hinder their performance.

To address these issues, Hochreiter and Schmidhuber [4] proposed the use of LSTM networks. LSTM networks are a variant of RNNs that use memory blocks, which consist of self-connected memory cells and three multiplicative units known as the input, output, and forget gates, to replace the hidden units in traditional RNNs. The gates allow for reading, writing, and resetting operations in the memory block and control the memory block’s behavior. This allows LSTM networks to better capture and retain long-term dependencies in sequential data, making them more effective at modeling complex temporal dynamics.

**Figure 4:**
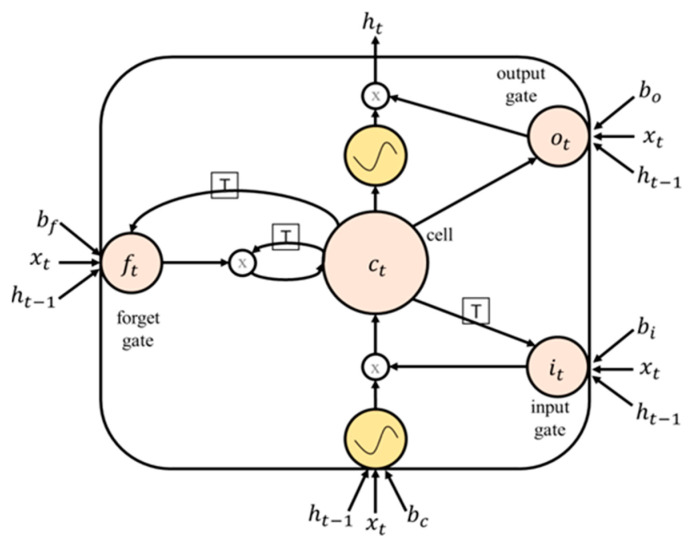
A basic single-cell LSTM.

The mathematical formulation of an LSTM unit can be described as follows (see Figure 4):

The input gate, i, controls whether or not to allow new information to enter the memory block. It is defined as follows:

(1) $$i=sigmoid\left(W_{ii}\;*\;x+W_{hi}\;*\;h+b_{i}\right)\;\;$$

where x is the input to the LSTM unit, h is the previous hidden state, $W_{ii}\;$and $W_{hi}$ are the weights connecting the input and previous hidden state to the input gate, and $b_{i}$ is the bias term for the input gate.

The forget gate, f, controls whether or not to erase the previous information stored in the memory block. It is defined as follows:

(2) $$f=sigmoid\left(W_{if}\;*\;x+W_{hf}\;*\;h+b_{f}\right)$$

where $W_{if}$ and $W_{hf}$ are the weights connecting the input and previous hidden state to the forget gate and $b_{f}$ is the bias term for the forget gate.

The output gate, o, controls whether or not to output the information stored in the memory block. It is defined as follows:

(3) $$o=sigmoid\left(W_{io}\;*\;x+W_{ho}\;*\;h+b_{o}\right)$$

where $W_{io}$ and $W_{ho}$ are the weights connecting the input and previous hidden state to the output gate and $b_{o}$ is the bias term for the output gate.

The memory cell, c, stores the information in the memory block. It is updated using the input and forget gates as follows:

(4) $$c^{\prime }=f\;*\;c+i\;*\;tanh\left(W_{ic}\;*\;x+W_{hc}\;*\;h+b_{c}\right)$$

where $W_{ic}$ and $W_{hc}$ are the weights connecting the input and previous hidden state to the memory cell and $b_{c}$ is the bias term for the memory cell.

The hidden state, h, is updated using the output gate and memory cell as follows:

(5) $$h=o\;*\;tanh\left(c^{\prime }\right)$$

These equations describe the basic operation of a single LSTM unit. Multiple LSTM units can be stacked together to form an LSTM network, which can be used for tasks such as dengue prediction by learning to model the temporal dynamics of the data.

#### 3.4.2. The Proposed LSTM

##### LSTM Architecture

This study developed and compared six different LSTM models for dengue prediction in Malaysia: LSTM, stacked LSTM (S-LSTM), LSTM with temporal attention (TA-LSTM), S-LSTM with temporal attention (STA-LSTM), LSTM with spatial attention (SA-LSTM), and S-LSTM with spatial attention (SSA-LSTM). 

**Figure 5:**
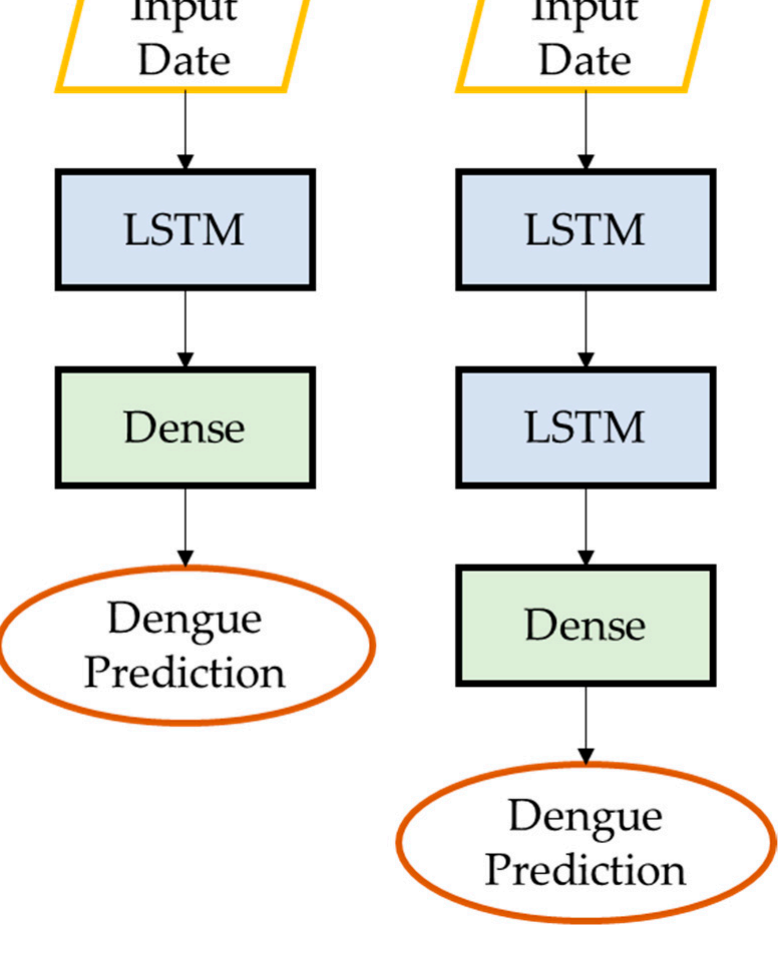
Architecture of LSTM and S-LSTM.

**Figure 6:**
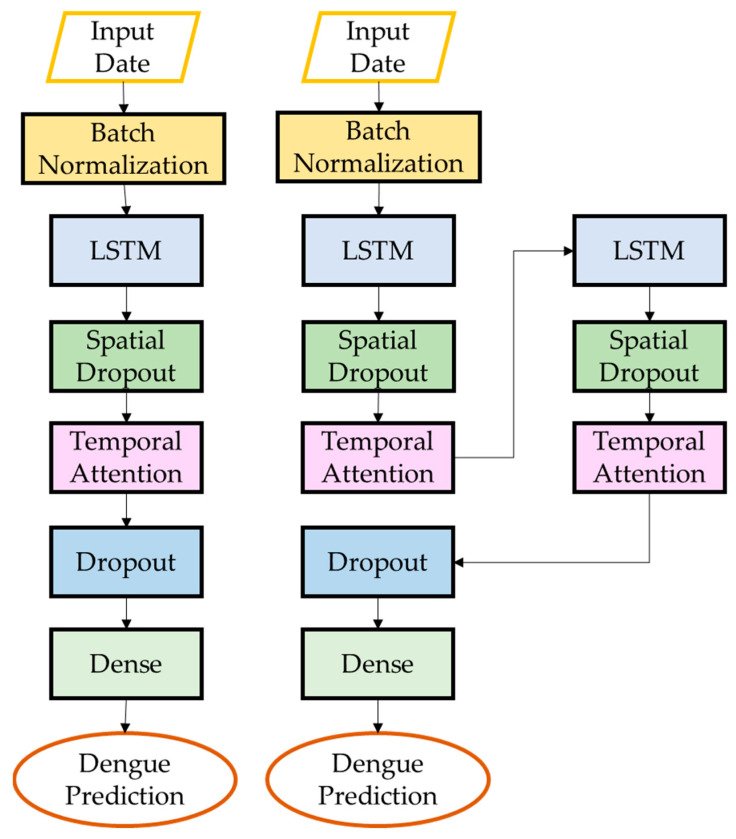
Architecture of TA-LSTM and STA-LSTM.

**Figure 7:**
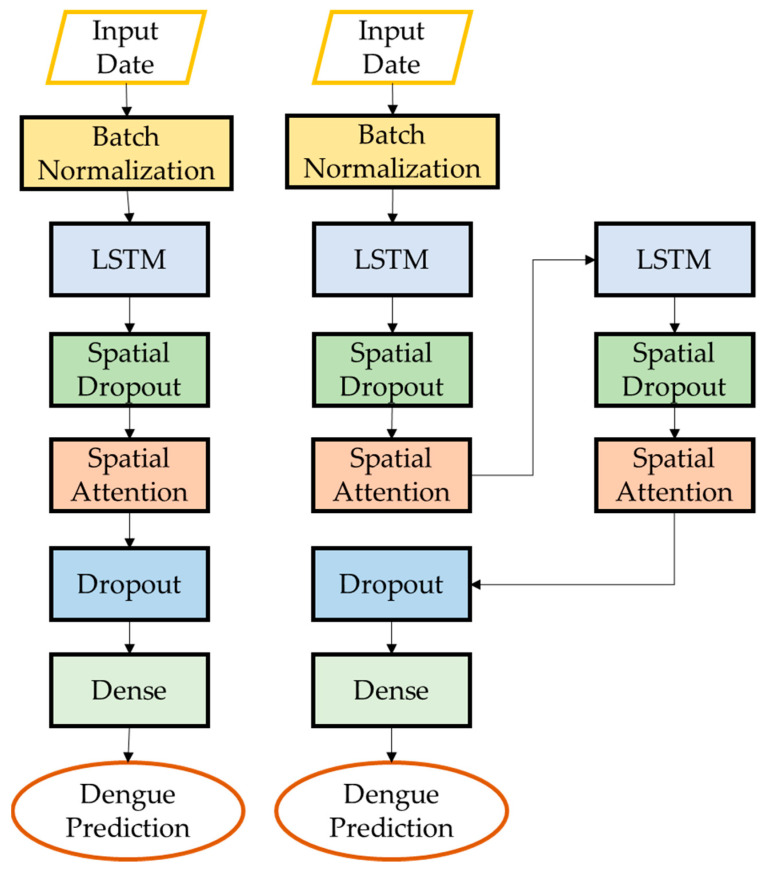
Architecture of SA-LSTM and SSA-LSTM.

The LSTM architecture consists of a single layer of LSTM units followed by a dense layer. The LSTM layer has 4 units and takes as input a 3D tensor with shape (*batch_size*, *look_back*, *n_features*), where *look_back* is the number of time steps to look back and *n_features* is the number of features in the input data. The dense layer has a single unit and produces a 2D tensor with shape (*batch_size*, 1) (Figure 5). The architecture of S-LSTM involved stacking two LSTM layers on top of each other, which can allow the model to capture more complex patterns in the data (Figure 5). TA-LSTM uses a temporal attention mechanism to weigh the input sequence at each time step, allowing the model to focus on the most important parts of the input when making predictions. Attention mechanisms can be helpful in situations where the input sequence is long and may contain a lot of noise or irrelevant information (Figure 6). STA-LSTM has the same architecture as TA-LSTM but with two LSTM layers stacked on top of each other (Figure 6). SA-LSTM is similar to TA-LSTM, but instead of attending to the input sequence over time, it attends to different parts of the input at each time step (Figure 7). This can be useful in cases where the input is a 2D spatial map and the model needs to focus on different parts of the map at different times. SSA-LSTM is a variant of LSTM that combines S-LSTM and spatial attention mechanisms (Figure 7). 

The architecture of all the models except LSTM included several other layers, including (1) a batch normalization layer that takes as input a 3D tensor with shape (*batch_size*, *look_back*, *n_features*). This layer normalizes the input data across the batch dimension, improving the model’s ability to learn. It also included a spatial dropout layer and dropout layers with a dropout rate of 0.2, which randomly sets a fraction of the input units to 0 during training, helping the model generalize to new data. 

The models were compiled using the ‘*mean_squared_error*’ loss function and the ‘*nadam*’ optimizer, with the *run_eagerly* parameter set to True. The ‘*mean_squared_error*’ loss function is a common choice for regression tasks, and the ‘*adam*’ optimizer is generally fast and memory-efficient. 

##### Spatial Attention Mechanism

Spatial attention in LSTM is a technique that allows the model to dynamically focus on different parts of the input data at different times, rather than processing all of the input equally. This can be useful for dengue prediction tasks, as it allows the model to selectively attend to the most relevant variables and features in the data, rather than being overwhelmed by noise or irrelevant information.

The spatial attention module in an LSTM network is typically implemented as a separate layer that sits between the input and the LSTM layers. The spatial attention module calculates a weighted sum of the input data based on a set of weights that are learned during training. These weights are determined by the model based on the importance of each input feature for the task at hand.

The mathematical formulation of the spatial attention module can be described as follows:

The input to the spatial attention module is typically a matrix X of size N×D, where N is the number of time steps and D is the number of input features.

The spatial attention module calculates a set of weights, a, for each input feature based on the input data and a set of learned parameters, W and b. The weights are calculated as follows:

(6) a=softmax(XW+b)

where softmax is a function that converts the input values to a probability distribution over the input features.

The weighted sum of the input data is then calculated as follows:

(7) $$X^{\prime }=Xa$$

This weighted sum represents the input data with the attention of the model focused on the most important features.

The weighted input is then passed through the LSTM layer as usual, and the hidden state is updated according to the equations described earlier. The output of the LSTM layer is then used to predict the dengue cases at the next time step.

Overall, the spatial attention module allows the LSTM network to selectively attend to the most relevant input features when making dengue predictions, which can improve the performance of the model.

##### Training Process

The training process of an LSTM model for dengue prediction involves iteratively adjusting the model’s parameters to minimize the difference between the predicted and actual dengue cases. The optimization algorithm used to adjust the parameters is an important factor that can influence the model’s performance and convergence speed. One commonly used optimization algorithm for LSTM models is *Adam*.

*Adam* is a stochastic gradient descent optimization algorithm that combines the advantages of two other popular optimization algorithms: root mean square propagation (*RMSprop*) and adaptive gradient algorithm (*AdaGrad*). It uses an exponentially decaying average of past squared gradients to scale the learning rate for each parameter, and an exponentially decaying average of past gradients to adjust the learning rate for each parameter. This allows *Adam* to automatically adapt the learning rate for each parameter based on its historical gradient, which can help the model converge more quickly and avoid becoming stuck in local minima.

The learning rate is a hyperparameter that controls the step size of the optimization algorithm. A smaller learning rate can help the model converge more slowly and reliably, but may also result in slower training times. A larger learning rate can speed up the training process, but may also cause the model to oscillate or diverge. In our dengue prediction model, we used a learning rate of 0.001, which is a commonly used default value that has been found to work well for many tasks. The loss function is a measure of the difference between the predicted and actual dengue cases. It is used to evaluate the model’s performance during training and guide the optimization process. The choice of loss function depends on the nature of the task and the characteristics of the data. For dengue prediction tasks, a suitable loss function might be the MSE, which measures the average squared difference between the predicted and actual dengue cases. The MSE loss is sensitive to outliers and punishes large errors more heavily than small errors, which can make it a useful choice for tasks involving continuous variables.

To train the LSTM models for dengue prediction, the steps presented in Procedure 1. Table 1 were applied:

#### 3.4.3. Benchmark Models

In this study, the performance of the proposed model was compared to several benchmark models to assess its relative effectiveness. The benchmark models included a support vector machine (SVM), a decision tree (DT), and an artificial neural network (ANN). Each of these models has been widely used in the literature for dengue prediction tasks and serves as a useful point of comparison for the proposed models.

The SVM model is a nonlinear version of the SVM model that uses a radial basis function kernel to handle more complex and nonlinear relationships. It is a powerful and robust model that can handle complex data but may be more computationally intensive to train and may require more careful tuning of its parameters. The SVM model was tuned using a grid search to find the optimal values of the hyperparameters C and gamma, which control the strength of the regularization and the width of the radial basis function kernel, respectively.

The DT model is a simple and efficient model that uses a tree structure to make predictions based on the values of the input features. It is a fast and interpretable model that is well suited for tasks involving large and complex datasets but may be prone to overfitting if the tree becomes too deep. The DT model was tuned using a grid search to find the optimal values of the hyperparameters related to the criterion for selecting the best split, the splitter, and the maximum tree depth.

The ANN model is a neural network with multiple hidden layers, which allows it to model more complex relationships and patterns in the data. It is a powerful and flexible model that is well suited for tasks involving large and complex datasets but may require more training data and may be more computationally intensive to train than simpler models. The ANN model was tuned using a grid search to find the optimal values of the hyperparameters related to the sizes of the hidden layers, the activation function, the optimizer, the learning rate, and the number of epochs.

For the SVM model, the hyperparameter *C* is set to 1.0. This value determines the strength of the regularization, with a larger value of *C* corresponding to less regularization. For the ANN model, the hyperparameter *hidden_layer_sizes* is set to a tuple with a single element, 100, indicating that the model has a single hidden layer with 100 neurons. The activation function is set to ‘*relu*’, the solver is set to ‘*adam*’, and the alpha regularization term is set to 0.0001. The *batch_size* is set to ‘auto’, the *learning_rate* is set to ‘*adaptive*’, the *learning_rate_init* is set to 0.001, and the *max_iter* is set to 200. The *early_stopping* flag is set to *True*, indicating that the model will use early stopping to terminate training when the validation score is not improving. For the DT model, the criterion is set to ‘*mse*’ (mean squared error), the splitter is set to ‘*best*’, the *max_depth* is set to 4, and the *max_features* is set to ‘*auto*’. The criterion determines the function used to measure the quality of a split, the splitter determines the strategy used to choose the split at each node, the *max_depth* determines the maximum depth of the tree, and the *max_features* determine the number of features to consider when looking for the best split.

#### 3.4.4. Evaluation Metrics

RMSE is a measure of the difference between the predicted values of a model and the actual values. It is calculated by taking the square root of the MSE, which is defined as the average of the squares of the differences between the predicted values and the actual values. The RMSE is a widely used metric for evaluating the performance of prediction models, as it provides a clear and intuitive sense of the overall error of the model.

First, the predicted values for a given set of input data using the model in question are determined. Then, these predicted values are compared to the actual dengue cases observed in the data, and the RMSE is calculated based on the differences between the two. The smaller the RMSE, the better the model is at predicting dengue cases.

RMSE is well suited for assessing dengue prediction models for several reasons. Firstly, it is a widely used and well-established metric that is easy to understand and interpret. Secondly, it is robust to outliers, meaning that it is not heavily influenced by extreme values that may be present in the data. Finally, RMSE is sensitive to the scale of the data, which is important when working with datasets that may have widely varying values. Overall, RMSE is a reliable and effective way to evaluate the performance of dengue prediction models and identify those that are most accurate and effective.

(8) $$RMSE=\sqrt{\frac{1}{N}\overset{N}{\underset{i=1}{\sum }}{\left(Y_{t}-{\underline{Y}}_{t}\right)}^{2}}$$

where $Y_{t\;}$is the dengue cases of observation for time t and ${\underline{Y}}_{t}$ is the number of cases predicted by the model. A smaller RMSE value indicates a smaller difference between the predicted and observed values and indicates a higher prediction performance of the model.

## 4. Results

### 4.1. LSTM Model Evaluation

Table 2 presents the comparison of different models, LSTM, S-LSTM, TA-LSTM, STA-LSTM, SA-LSTM, and SSA-LSTM. The main difference between these models is the type of attention mechanism being used, with some models using temporal attention (TA-LSTM, STA-LSTM) and others using spatial attention (SA-LSTM, SSA-LSTM). The LSTM and S-LSTM models do not use any attention mechanism. The models were trained and evaluated on a dataset of monthly dengue cases in Malaysia from 2010 to 2016. The task being performed was the prediction of the number of dengue cases based on various climate, topographic, demographic, and land-use variables.

The lookback indicates the number of time steps that the models are considering when making predictions. When comparing the models by looking at the minimum, maximum, average, and standard deviation values, the SSA-LSTM model has the lowest average error (3.17 RMSE), while the LSTM model has the highest average error (4.15 RMSE). Looking at the results in the table, it seems that the models with stacked LSTM layers generally perform better than the other models, regardless of the use of attention mechanisms. This may be because these models have more parameters and can capture more complex patterns in the data.

**Figure 8:**
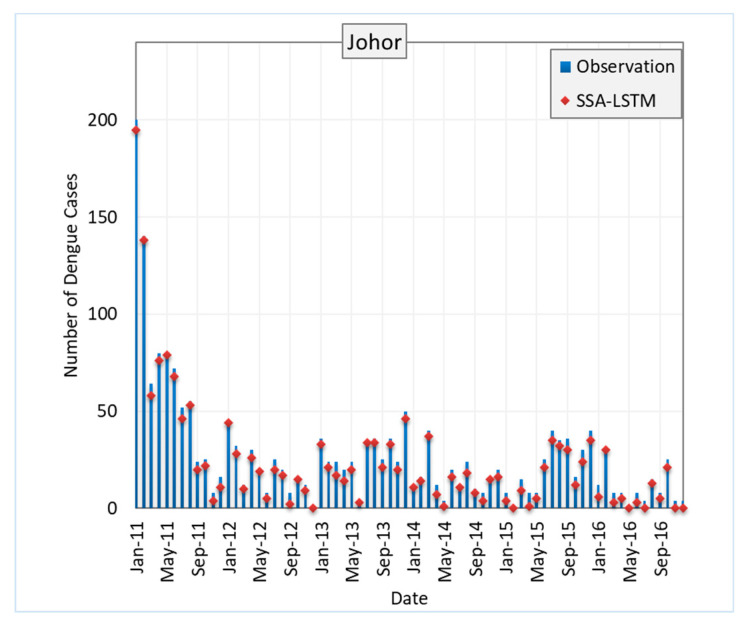

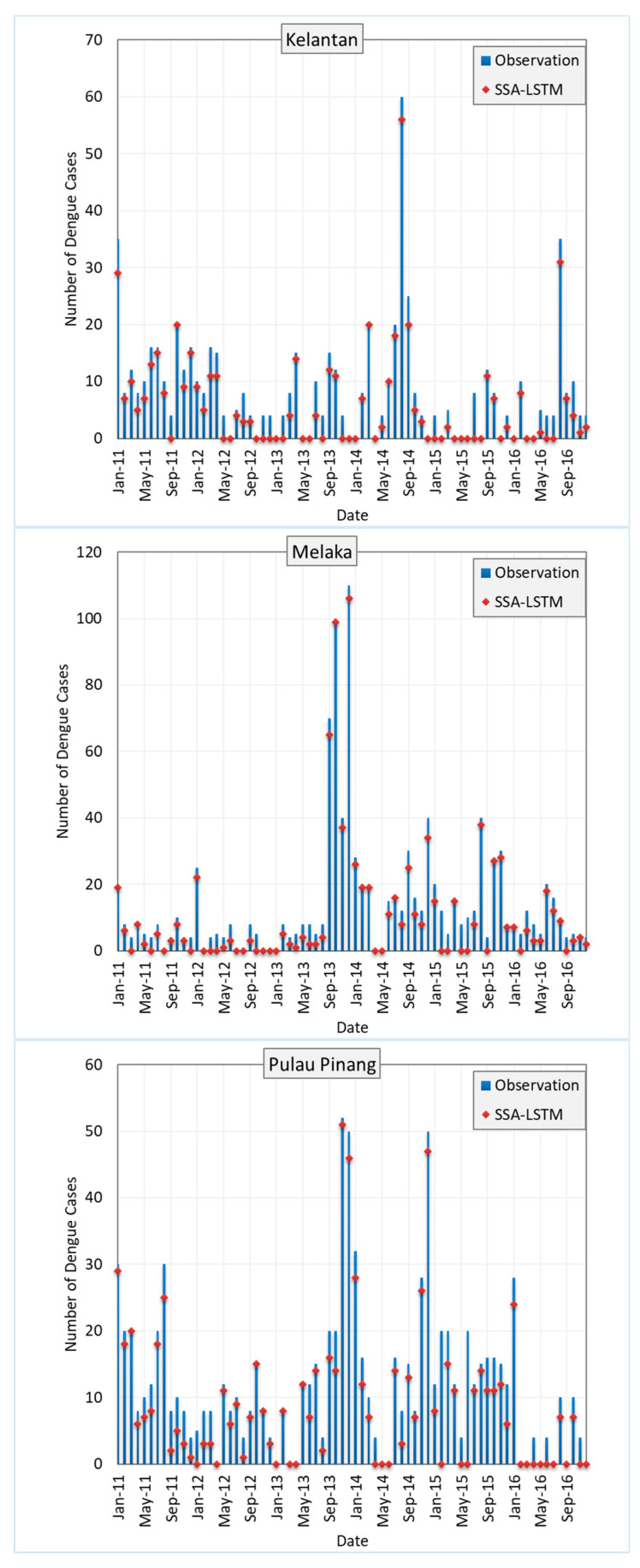

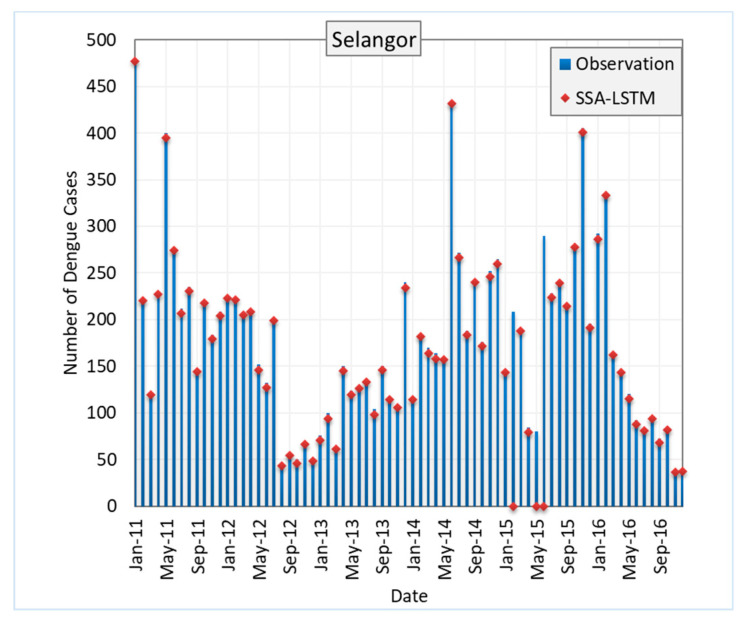
Performance of SSA-LSTM model for dengue fever prediction in five Malaysian states, measured by RMSE.

**Figure 9:**
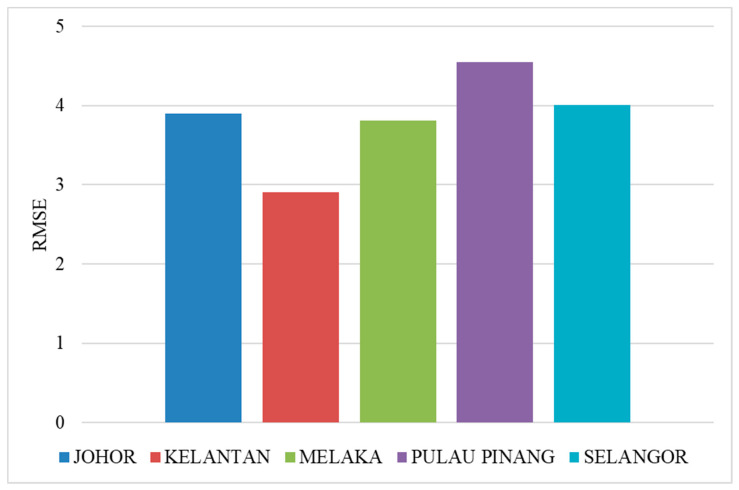
Average performance of SSA-LSTM model for dengue fever prediction in five Malaysian states, measured by RMSE.

Figure 8 shows the RMSE of SSA-LSTM models for different states in Malaysia in comparison to the actual dengue cases in the states. The RMSE is a measure of the difference between the predicted values and the true values, with a lower RMSE indicating a better model performance. In general, the SSA-LSTM models perform relatively well, with RMSE values ranging from 2.91 to 4.55. The state with the lowest RMSE is Kelantan, followed by Melaka, Johor, Selangor, and Pulau Pinang. This indicates that the SSA-LSTM models perform better in Kelantan and Melaka compared to the other states (Figure 9).

It is worth noting that the RMSE values are generally lower than 5, which suggests that the SSA-LSTM models are able to make relatively accurate predictions. However, there is still room for improvement, as a lower RMSE would indicate a better model performance. Further analysis of the data and the models may be needed to understand the reasons behind the differences in performance across the states and to identify ways to improve the model performance. More details are given in the Section 4.4. 

### 4.2. Comparison with Benchmark Models

Table 3 compares the performance of four different models—SVM, DT, ANN, and SSA-LSTM—in a prediction task using different lookback periods, measured by RMSE. The lookback periods range from 1 to 6 months. Overall, the SSA-LSTM model performs the best, with an average RMSE of 3.17 across all lookback periods. This is significantly lower than the average RMSEs of the other three models, which are 4.59 for SVM, 5.43 for DT, and 4.63 for ANN. The SSA-LSTM model performs particularly well with lookback periods of 4 and 5 months, with RMSEs of 2.71 and 2.65, respectively. However, its performance degrades slightly as the lookback period decreases, with RMSEs of 3.58 for a lookback period of 3 months and 3.07 for a lookback period of 2 months. In comparison, the performance of the other three models is more consistent across different lookback periods, with relatively stable RMSE values.

### 4.3. Impact of Spatial Attention Mechanism

When comparing temporal and spatial attention LSTM models with different lookback periods, it is clear that the spatial models generally perform better at predicting dengue cases. The average RMSEs for the two temporal models, TA-LSTM and STA-LSTM, are 4.13 and 3.67, respectively, while the average RMSEs for the two spatial models, SA-LSTM and SSA-LSTM, are 3.87 and 3.17, respectively. The SSA-LSTM model performs particularly well, with the lowest RMSE of any model at all lookback periods. In general, temporal attention mechanisms are designed to allow a model to focus on specific parts of the input sequence over time, while spatial attention mechanisms allow a model to focus on specific parts of the input. These mechanisms can be helpful in situations where the input sequence is long and may contain a lot of noise or irrelevant information, or where the input is a 2D spatial map and the model needs to focus on different parts of the map at different times.

At shorter lookback periods, the performance of the spatial models is particularly strong. For example, at a lookback period of 1 month, the SSA-LSTM model has an RMSE of 3.22, while the SA-LSTM model has an RMSE of 3.65. In comparison, the TA-LSTM model has an RMSE of 3.72 and the STA-LSTM model has an RMSE of 3.26. This trend continues at a lookback period of 2 months, with the SSA-LSTM model performing the best with an RMSE of 3.07, followed by the SA-LSTM model with an RMSE of 3.06.

As the lookback period increases, the performance of all the models tends to degrade. However, the spatial models still perform better than the temporal models. For example, at a lookback period of 3 months, the SSA-LSTM model has an RMSE of 3.58, while the SA-LSTM model has an RMSE of 4.95. In comparison, the TA-LSTM model has an RMSE of 5.11 and the STA-LSTM model has an RMSE of 4.55. This trend continues at a lookback period of 4 months, with the SSA-LSTM model performing the best with an RMSE of 2.71, followed by the SA-LSTM model with an RMSE of 3.85.

Overall, the use of spatial attention appears to be a key factor in improving prediction performance, especially at shorter lookback periods. The SSA-LSTM model, which uses both spatial attention and two LSTM layers, performs particularly well, with the lowest RMSE of any model at all lookback periods.

### 4.4. Discussion

The results of the comparison show that the SSA-LSTM model performs the best, with the lowest average RMSE of 3.17. This model uses both spatial and temporal attention mechanisms, which may be effective at capturing relevant patterns in the data and making more accurate predictions. It is also worth noting that the models with stacked LSTM layers generally perform better than the other models, regardless of whether they use attention mechanisms or not. This suggests that these models may be more effective at capturing complex patterns in the data and making more accurate predictions. It is important to note that the results of this comparison may not necessarily generalize to other datasets or tasks. It would be necessary to evaluate these models on other datasets and tasks to confirm their relative performance in those contexts.

There are a few potential reasons why the LSTM models with attention mechanisms and stacked LSTM layers performed better than the other models in this comparison. One reason could be that these models can more effectively process the complex and varied set of input variables, such as climate, topographic, demographic, and land-use variables, which may all be relevant for predicting dengue cases. The attention mechanisms may also be effective at identifying which of these variables are most important for making predictions at each time step, which could improve the accuracy of the model.

From an epidemiological perspective, the results of this comparison suggest that LSTM models with attention mechanisms and stacked LSTM layers may be effective at predicting the number of dengue cases in Malaysia based on various factors that may influence the transmission of the disease. For example, climate variables such as temperature and humidity can affect the survival and reproduction of the mosquitoes that transmit dengue, while topographic variables such as the presence of bodies of water can influence the distribution of mosquito breeding sites. Demographic variables such as population density and land-use variables such as the amount of urbanization can also affect the transmission of dengue.

This research showed that LSTM with spatial attention can be a powerful approach for predicting dengue fever. LSTM networks are well suited for modeling time series data and can capture patterns that span multiple time steps. This can be particularly useful for predicting dengue fever, which may be influenced by both short-term and long-term factors. The use of spatial attention allows the model to weigh different features differently when making predictions. This can be useful for focusing on the most relevant features, such as environmental factors or population density, which can improve the accuracy of the model. LSTM networks can handle missing data more effectively than some other models, as they can retain information from previous time steps. This can be useful in situations where data may be missing or incomplete. 

However, there are also some potential limitations to using LSTM with spatial attention for dengue fever prediction. LSTM networks can be complex and require a large amount of data and computational resources to train effectively. This can make them more challenging to implement and may limit their practical applicability in some cases. LSTM networks can be difficult to interpret, as they are black-box models that do not provide insight into the specific features or patterns that they are using to make predictions. This can make it difficult to understand how the model is making decisions and to identify any potential biases in the data. 

One potential limitation of the dataset used in this research is that it only includes dengue case data at the state level in Malaysia and does not include data at a more granular level, such as at the district or city level. This may limit the ability of the models to capture local variations in dengue prevalence and to identify specific hotspots or areas at higher risk of outbreaks. Additionally, the dataset only includes dengue case data from 2010 to 2016, which may not be sufficient to capture longer-term trends or patterns in the disease. This could potentially limit the models’ ability to accurately predict dengue cases in future years. Another limitation of the dataset is that it only includes a few variables related to climate, geography, and demographics and does not include other potentially relevant factors such as data on vector control measures or population mobility. This could potentially limit the models’ ability to accurately capture the relationships between these variables and dengue cases. These limitations of the dataset may have affected the results of the study in several ways. First, the limited scope of the data may have limited the models’ ability to accurately capture the relationships between the input variables and dengue cases. This could have resulted in lower model performance and less accurate predictions. Second, the lack of data on more granular spatial scales or longer periods may have limited the models’ ability to capture longer-term trends or patterns in the data, which could have affected the models’ performance and the accuracy of the predictions. Finally, the limited number of input variables may have reduced the models’ ability to capture complex relationships between the variables and dengue cases, which could have led to lower model performance and less accurate predictions.

In addition, there would be a potential impact of climate change on the precision of the models’ predictions. As climate patterns shift, the effects of variables such as rainfall and land surface temperature may change over time, which could affect the accuracy of our models. Therefore, ongoing monitoring and updating of the models will be necessary to ensure that they remain reliable in the face of changing environmental conditions. Future studies could also explore the potential for incorporating additional climate-related variables into the models to further improve their predictive capabilities in the context of climate change.

The use of LSTM with spatial attention for dengue fever prediction can have practical implications for public health efforts to prevent and control dengue fever outbreaks. Some potential applications of this approach are as follows:

○ Early warning systems: Predictive models based on LSTM with spatial attention can be used to identify areas at high risk for dengue fever outbreaks. This information can be used to implement targeted prevention measures, such as mosquito control efforts, and can help to reduce the impact of outbreaks on affected communities.
○ Resource allocation: Predictive models can be used to identify areas where resources (e.g., healthcare staff, medical supplies) may be needed in the event of a dengue fever outbreak. This can help public health officials to allocate resources in a targeted and efficient manner.
○ Surveillance and monitoring: Predictive models can be used to monitor the risk of dengue fever outbreaks over time, allowing public health officials to identify trends and changes in the risk of outbreaks. This can help to inform the development of prevention and control strategies.

Overall, the use of LSTM with spatial attention for dengue fever prediction can help to improve the effectiveness of public health efforts to prevent and control outbreaks of the disease. It is important to carefully consider the limitations of this approach, such as the need for large amounts of data and computational resources, and to consider the specific needs and resources of the communities where the model will be applied.

## 5. Conclusions

The main contribution of the study was the development and comparison of several LSTM models for dengue prediction, and the finding that the SSA-LSTM model has the best performance among the compared models. This suggests that the use of attention mechanisms and stacked LSTM layers can be effective in improving the accuracy of dengue prediction models. The proposed model contributes to the field of dengue fever prediction by providing a new approach for modeling and predicting dengue cases. The use of an LSTM model, which is designed to capture long-term dependencies in time series data, allows the model to consider the past values of the dengue cases when making predictions. The use of spatial attention, which allows the model to focus on specific input features or regions, can help the model to better capture the relationships between the input variables and the dengue cases. Overall, the proposed deep learning LSTM model with spatial attention provides a new tool for understanding and predicting dengue cases, and its effectiveness suggests that this approach has the potential to be a valuable addition to the existing body of knowledge in the field.

For future works, there are still many research problems to tackle, including using transfer learning to train a model on dengue data from one geographic region and then fine-tune it for prediction in a different region; incorporating expert knowledge or domain-specific information into the model design, e.g., incorporating expert-defined rules or incorporating data on the presence of mosquito breeding sites; exploring other types of attention mechanisms, such as multi-head attention or self-attention, to see if they improve model performance; evaluating the performance of the models on different time scales, such as weekly or daily dengue case counts, to see if the results vary depending on the granularity of the data; and using model distillation or compression techniques to reduce the complexity of the model while maintaining good performance for dengue prediction, which can make it easier to interpret.

## Figures and Tables

**Table 1:** Procedure 1.

1	Prepare the training data by preprocessing and formatting them for input into the model. This might involve scaling the data to a common range, handling missing values, and creating lag variables to capture the temporal dynamics of the data.
2	Initialize the model’s parameters and set the optimization algorithm, learning rate, and loss function.
3	Iterate through the training data in mini-batches. For each mini-batch, pass the input data through the LSTM model and use the predicted dengue cases to calculate the loss according to the chosen loss function.
4	Use the Adam optimization algorithm to adjust the model’s parameters based on the calculated loss and the learning rate.
5	Repeat steps 3 and 4 for a set number of epochs, or until the loss reaches a satisfactory level or stops improving.
6	Monitor the training process by evaluating the model’s performance on a validation set, which is a subset of the training data that is used to assess the model’s generalization performance. This can help identify overfitting and prevent the model from learning from noise in the training data.
7	Save the trained model and its parameters for later use.
8	Test the trained model on a separate test set, which is a subset of the data that has not been used in training or validation. This allows us to evaluate the model’s performance on unseen data and gauge its real-world effectiveness.

**Table 2:** Performance of different LSTM models for dengue prediction in Malaysia.

Lookback	Model					
	LSTM	S-LSTM	TA-LSTM	STA-LSTM	SA-LSTM	SSA-LSTM
1	3.75	3.72	3.72	3.26	3.65	3.22
2	3.57	3.62	3.62	3.12	3.06	3.07
3	5.06	5.11	5.11	4.55	4.95	3.58
4	3.74	3.85	3.85	3.42	3.85	2.71
5	3.99	3.85	3.85	3.36	3.62	2.65
6	4.78	4.62	4.62	4.31	4.11	3.76
Min	3.57	3.62	3.62	3.12	3.06	2.65
Max	5.06	5.11	5.11	4.55	4.95	3.76
Average	4.15	4.13	4.13	3.67	3.87	3.17
Std.	0.61	0.59	0.59	0.6	0.58	0.41

**Table 3:** Performance of the proposed model (SSA-LSTM) compared to three benchmark methods, namely SVM, DT, and ANN.

Lookback	Model			
	SVM	DT	ANN	SSA-LSTM
1	4.44	4.82	4.66	3.22
2	4.37	5.58	4.42	3.07
3	4.76	5.6	4.53	3.58
4	4.44	5.93	4.42	2.71
5	4.67	5.69	4.76	2.65
6	4.86	4.96	5.00	3.76
Min	4.37	4.82	4.42	**2.65**
Max	4.86	5.93	5.00	**3.76**
Average	4.59	5.43	4.63	**3.17**
Std.	**0.20**	0.43	0.22	0.41

## Data Availability

Data supporting reported results can be available upon reasonable request from the corresponding author.
